# Processing and Properties of Single-Crystal Copper Wire

**DOI:** 10.3390/mi14112080

**Published:** 2023-11-10

**Authors:** Jun Cao, Xuefeng Wu, Chenghao Su, Hewei Jia, Yongzhen Sun

**Affiliations:** School of Mechanical and Power Engineering, Henan Polytechnic University, Jiaozuo 454003, China; wuxuefeng@hpu.edu.cn (X.W.); sumurong666@gmail.com (C.S.); 212105020051@home.hpu.edu.cn (H.J.); syz@home.hpu.edu.cn (Y.S.)

**Keywords:** copper wire, multipass drawing, deformation, heat treatment

## Abstract

The effects of drawing parameters and annealing process on the properties and microstructure of single crystal copper wire are studied using a wire-drawing machine, heat-treatment equipment, microcomputer-controlled electronic universal tester, resistance tester, and scanning electron microscope. The results show that, after drawing the single-crystal copper wire with a single-pass deformation of 14%, the grains elongate along the tensile direction, tensile strength increases from 500.83 MPa to 615.5 Mpa, and resistivity changes from 1.745 × 10^−8^ Ω·m to 1.732 × 10^−8^ Ω·m. After drawing at a drawing rate of 500 m/min, the degree of grain refinement increases and tensile strength increases from 615.5 Mpa to 660.26 Mpa. When a copper wire of Φ0.08 mm is annealed, its tensile strength decreases from 660.26 Mpa to 224.7 Mpa, and elongation increases from 1.494% to 19.87% when the annealing temperature increases to 400 °C. When the annealing temperature increases to 550 °C, the tensile strength and elongation decrease to 214.4 MPa and 12.18%, respectively.

## 1. Introduction

Wire bonding occupies a dominant position in the field of chip packaging because of its mature technology and low cost [1,2,3,4]. Wire bonding can connect the metal cloth welding area or microelectronic packaging I/O lead to the semiconductor chip welding area, which is an important process link in the semiconductor packaging process. Its construction quality has a great influence on the functional applications of the semiconductors. Compared to gold wire, bonded copper wire has a lower production cost, good conductivity, and superior mechanical properties which facilitates its widespread use in many applications such as semiconductor packaging, integrated circuits, and light-emitting diodes [5,6,7]. Although pure copper has excellent electrical and thermal conductivity and corrosion resistance, its strength and hardness are low. Improving its mechanical properties without reducing the electrical conductivity is one of the key challenges in the preparation of ultrafine and high-performance copper wires [8,9,10].

Integrated circuits and electronic components are progressing towards miniaturization and high integration, and the demand for ultrafine, high-performance copper bonding wires with diameters less than 30 μm is increasing. Song et al. studied the variations in the mechanical properties and electrical conductivity of Φ0.02 mm Cu-1Ag alloy wire with strain. They concluded that with the increase in strain, the tensile strength of the Cu-1Ag alloy increased, whereas the electrical conductivity first decreased and then stabilized [11]. Zhu et al. prepared Φ0.04 mm Cu-4wt% Ag alloy wire by continuous directional solidification and drawing. The evolution of the microstructure and properties at each stage of the drawing was studied. The tensile strength, yield strength, and electrical conductivity of the alloy were 1048 MPa, 886 MPa, and 75.2% IACS (IACS: international annealed copper standard), respectively, after deformation at a tensile of 11.28 [12]. Gokhfel’d et al. studied the microstructure, mechanical properties, and electrical conductivity of a Cu3Pd alloy after severe cold plastic deformation following repeated stretching and annealing, and determined the microstructure evolution of the alloy after severe deformation and subsequent annealing [13]. Fu et al. tested the strength and electrical conductivity of Ag-11.40Cu-0.66Ni-0.05Ce (wt%) alloy wire at different deformation stages and observed the microstructure during deformation. After a large deformation drawing, the room-temperature Vickers hardness of the as-cast alloy increased from HV 81.6 to HV 169.3, and the electrical conductivity increased from 74.3% to 78.6% IACS [14]. Cheng et al. studied the evolution of the microstructure, mechanical properties, and electrical properties of Cu-20wt% Ag alloy wire during the drawing process. It was concluded that when the diameter was stretched to 0.02 mm (*η* = 11.94), the tensile strength of the alloy was 1682 MPa and elongation was 2.0%. The relationship between the tensile strength, elongation, and diameter conformed to Allometric and Boltzmann functions, respectively [15]. Cao studied the performance and microstructure changes in Ag-4Pd alloy wire during heat treatment and determined the best heat treatment temperature for achieving excellent wire performance [16]. Cheng et al. studied the dislocation boundary of single-crystal copper wire. In the spatial distribution of a high-angle dislocation boundary, when the strain is greater than 2.77, the dislocation boundary extends from the center to the surface with the increase in strain [17]. By studying the texture evolution of single-crystal copper wire when the strain is less than 4.12, the results show that when the initial orientation of single-crystal copper is <111>, <100>, and <110>, the grains are refined during cold drawing. A mixture of <111> and <100> fiber textures is formed under high strain [18]. Li et al. studied that a single-crystal copper wire with a diameter of 1 mm was drawn into 0.2 mm and analyzed its structure and properties. The results show that fine grain strengthening and dislocation strengthening are the key factors affecting the strength and conductivity of single-crystal copper wire [19]. At present, there are many studies on most alloy wires at home and abroad but few studies on fine single-crystal copper wires, and the tensile strength of single-crystal copper wires discussed in the existing literature is low. This paper takes single-crystal copper wire as the research object, studies the deformation behavior of copper wire under different single-pass deformation and different drawing speeds, analyzes the influence of drawing parameter change on the copper wire performance, and improves the theoretical system of drawing parameter change on the copper wire drawing process. The properties and microstructure differences of copper wire under different drawing conditions and different heat treatment conditions are studied. The tensile strength of copper wire also reached 660 MPa. After annealing, the strength reaches 225 MPa and the elongation reaches 20%.

## 2. Original Materials and Experimental Details

### 2.1. Original Materials and Equipment

**Figure 1:**
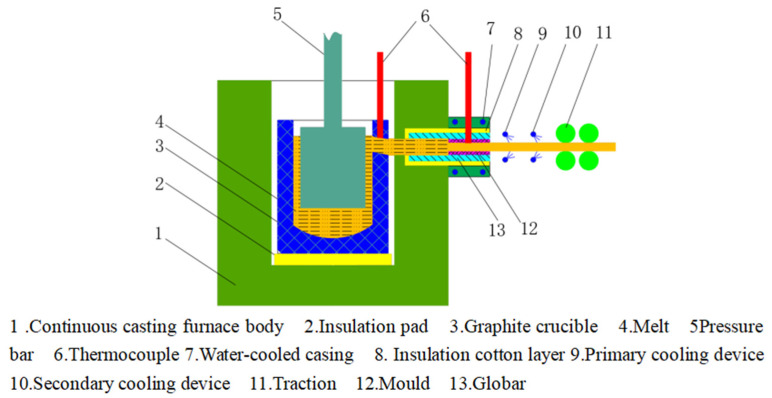
Schematic diagram of heated-mold continuous casting device.

The original material was a single-crystal copper rod with a diameter of 8.0 mm, prepared by a thermal horizontal continuous casting equipment. The single-crystal copper rod with a diameter of 8.0 mm was drawn to a diameter of 3.0 mm by a large diameter wire drawing machine, and then multi-mode drawn by a medium diameter wire drawing machine to a diameter of 0.9 mm. Finally, the single-crystal copper wire with a diameter of 0.2 m was processed by multimode drawing of a small diameter wire drawing machine. The single-crystal copper samples were prepared on a self-made hot horizontal continuous casting equipment, which is presented in Figure 1. First, the electrolytic copper raw material with a mass fraction of 99.95% was added to the graphite crucible. The temperature of the copper liquid was 1140~1250 °C, and the smelting process was protected using an inert gas. After the raw material melted completely, the pressure bar pressed the melt in the graphite crucible downward so that the melt entered the mold through the guide tube. While pouring, the traction rod with a groove at the front end entered the mold and contacted the melt. The pouring temperature was 1080~1110 °C, the traction speed was 40 mm/min, the cooling water temperature was 20 °C, the flow rate was 25 mm/min, and the cooling distance was 10 mm. Under these conditions, the melt changed from liquid to solid, producing a single-crystal copper rod blank of 16 mm. The single-crystal copper exhibited highly oriented longitudinal grains with a low number of grain boundaries, which reduced the hindrance to dislocation movement and significantly improved the plastic deformation ability.

The raw material used in the experiment was a 4 N single-crystal copper wire (single-crystal copper with purity 99.99%). The wire possessed original parameters of a tensile strength measuring 500.83 MPa, an elongation of 0.93%, and a resistivity measuring 1.745 × 10^−8^ Ω·m. An LH150-36-type very fine wire drawing machine was used to draw the single-crystal copper wire. A KD2-0.02-type electronic universal tester and an HS-3004A tensile tester were used to test the mechanical properties of the copper wire; the specimen length was 100 mm, and the tensile speed was 10 mm/min. The electrical properties were tested by an SB2231-type resistance tester, and the length of the specimen was 1000 mm. In the heat treatment test of the 0.08 mm single-crystal copper wire, the SD-40 continuous heat treatment equipment was utilized. The heat treatment process involved the use of electric heating as the heating mode, a quartz glass annealing tube with a length of 1200 mm, and a temperature fluctuation range of 2 °C. An angular displacement sensor was employed to control the wire tension during the heat treatment, with the tension range set between 0.01–0.10 N for various annealing rate tests. The annealing rate, which refers to the speed at which the wire passes through the annealing tube, represents the closing speed of the annealing heat treatment.

Metallographic preparation was carried out as follows, by placing the intercepted copper wire strands into the bottom of a rubber mold and pouring epoxy resin to solidify naturally. The polishing process involved the use of 600#, 800#, 1000#, 1200#, and 2000# SiC sandpaper on a polishing machine. Throughout the process, the surface of the sandpaper was continuously rinsed with water flow. To ensure a thorough sanding operation, the specimen was rotated by 90° after each stage of sanding, with each stage carried out without any remnants of the previous scratch on the surface. Following the grinding steps, the surface of the specimen exhibited no apparent scratches. Subsequently, rough polishing and fine polishing were executed using 1.0 μm and 0.5 μm diamond suspension spray, resulting in a smooth specimen surface without any visible scratches under microscope observation. The corrosion solution was a mixture of 5 g of copper chloride and 100 mL of ammonia solution, and the corrosion solution was ready for use. The corrosion time was 5–10 s; after completion of the corrosion, we used distilled water or anhydrous ethanol cleaning and blow-dried the specimen. The surface of the corrosion cleaned specimen was sprayed with gold and then put under the scanning electron microscope of JEOL JSM-6700F to analyze and observe the changes in microstructure under different conditions. Scanning electron microscope manufacturer is Nippon Electron Co. (Huizhou, China).

### 2.2. Test Scheme

To investigate the effects of single-pass deformation on the mechanical and electrical properties of copper wire, a multipass drawing test was conducted on the original Φ0.25 mm copper wire. Three experimental schemes were devised, as shown in Table 1, where the single-pass drawing amounts were set at 18%, 14%, and 10%, respectively. The drawing speed was maintained at 500 m/min, resulting in a final wire diameter of Φ0.135 mm. At each pass, samples were collected and tested for their tensile strength, elongation, and resistivity. Subsequently, the effects of the three different levels of single-pass deformation on the properties of the copper wire were compared and analyzed.

Considering the influence of electrical conductivity, the Φ0.135 mm copper wire with a single-pass drawing amount of 14% (Scheme 2 of Table 1) was selected for the drawing test at three drawing rates, as shown in Table 2. The three schemes employed drawing speeds of 300, 500, and 700 m/min and deformation of a single pass of 15%, resulting in a wire with a diameter of Φ0.08 mm. The samples were tested for tensile strength, elongation, and resistivity to compare the effects of the three drawing rates on the mechanical properties of copper wire.

The drawing speed was set at 500 m/min and a wire of Φ0.08 mm was annealed at 250, 300, 350, 400, 450, 500, and 550 °C. The annealing speed was 100 m/min for the heat treatment test, as shown in Table 3. The samples underwent measurements of tensile strength, elongation, and resistivity, and the changes in mechanical and electrical properties at different annealing temperatures were studied. Metallographic samples of the wires annealed at different temperatures were fabricated, and their microstructure evolution was observed using a scanning electron microscope (SEM). The evolution of the microstructure of the material under different temperature conditions was analyzed.

The Φ0.08 mm wire rod was selected with a drawing speed of 500 m/min. As shown in Table 3, the wire rod was annealed at rates of 20, 40, 60, 80, 100, and 120 m/min. The annealing temperature was 300 °C. The tensile strength, elongation, and resistivity of the aforementioned copper wire rod were measured, and the material properties were studied at different annealing rates.

## 3. Test Results and Analysis

### 3.1. Effect of Different Deformation on the Microstructure of Single-Crystal Copper Wire

**Figure 2:**
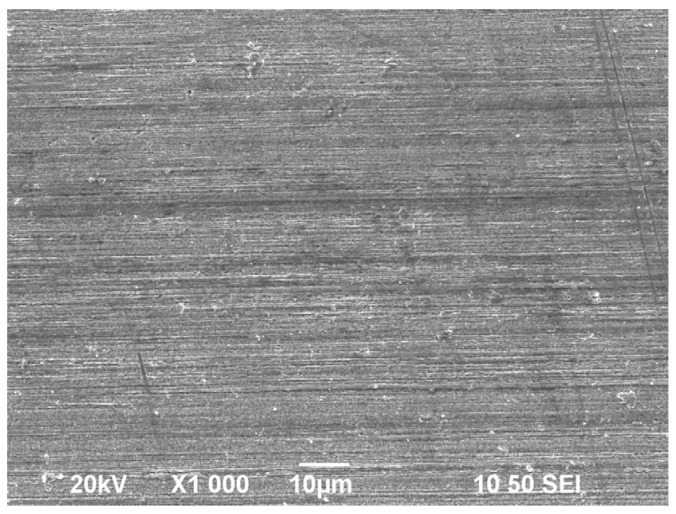
Microstructure of original copper wire.

In the drawing process of single-crystal copper, the slip phenomenon occurs first, and each grain is elongated along the drawing direction. After that, the columnar crystal inside the wire is broken. The wire changes from the single-crystal state to a polycrystalline state, and the dislocation density increases. With the increase in strain, the broken grains gradually become orderly and elongate along the drawing direction; the degree of grain refinement continues to increase, finally forming a fibrous structure. The microstructure of the single-crystal copper wire after drawing with different single-pass deformations is as follows. Figure 2 is the SEM image of the Φ0.25 mm sample. After a single pass of deformation of 18%, 14%, and 10%, the copper wires all have similar fiber structures, and there is no obvious difference.

### 3.2. Effect of Deformation on Mechanical and Electrical Properties of Single-Crystal Copper Wire

With the increase in the strain rate, the dynamic recovery process of the dislocations is inhibited, and the work-hardening effect is higher than that of dynamic recovery. Therefore, dislocations are accumulated more at high strain rates of deformation, thus improving the strength of the material. At the same time, the dynamic recovery of dislocations is a thermal activation process. High-temperature deformation can accelerate the dynamic recovery of dislocations, thus reducing the dislocation density. The low temperature and high strain rate inhibit the dynamic recovery process of dislocations so that dislocations can be further accumulated and the work-hardening rate of the material can be improved. Therefore, during the cold drawing of single-crystal copper wire, low-temperature high-strain-rate deformation is beneficial for the formation of twins. The grain boundary and twin boundary hinder the slip of dislocations simultaneously; thus, dislocations are entangled at these interfaces, which increases the strength of the material.

**Figure 3:**
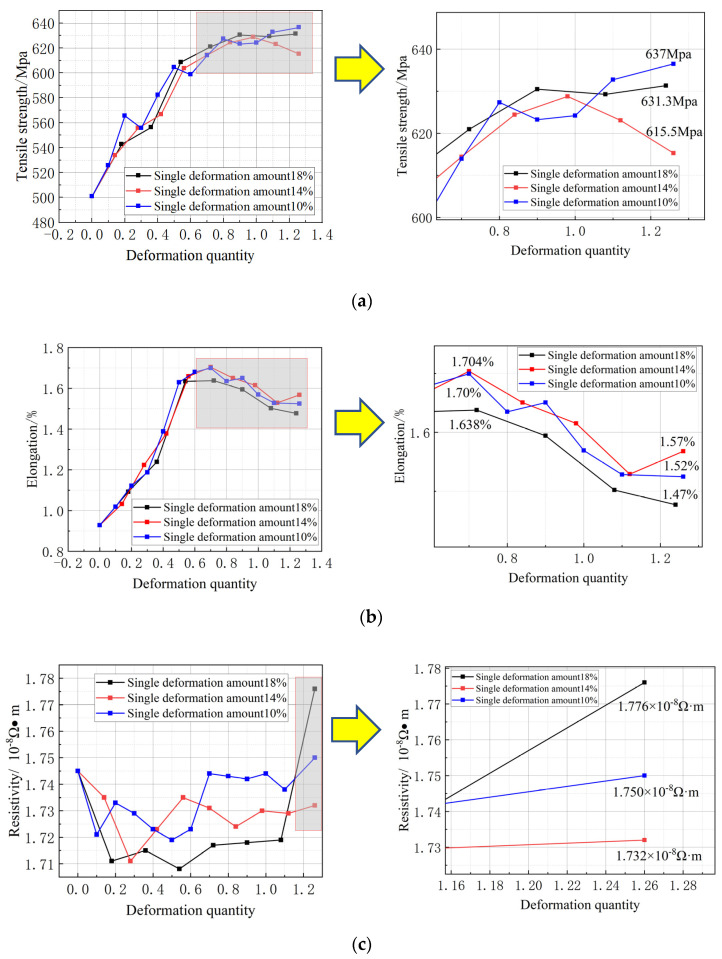
Mechanical properties and electrical conductivity of single-crystal copper wires under different deformations. (**a**) Tensile strength change in copper wire; (**b**) elongation change in copper wire; (**c**) resistivity change in copper wire.

Figure 3a is the change curve of the tensile strength of the copper wire under three different strains. As the strain increases, the tensile strength of the wire initially rises from 500.83 MPa to 631.3 MPa, 615.5 MPa, and 637 MPa, before stabilizing. This is due to lattice distortion during deformation, which elongates and refines the grain. After grain refinement, the grain boundary area, slip effect on dislocation, and the deformation-resistance index (tensile strength, etc.) of the metal increases with the increase in the deformation degree.

Figure 3b is the change curve of elongation after drawing at three different deformations. The elongation increases from 0.93% to 1.47%, 1.57%, and 1.52%. When the strain is approximately 0.7, the elongation reaches the maximum value (1.638%, 1.704%, and 1.70%). Then, with the increase in the deformation degree, the elongation gradually decreases because of the occurrence of intragranular and intergranular damage during deformation, uneven deformation, etc., which is the plastic index (elongation, etc.) of the metal.

Figure 3c shows the change curve of resistivity of the single-crystal copper wire in different deformation tests. The final result shows that the resistivity of the wire with 18% deformation is the highest at 1.776 × 10^−8^ Ω·m. The wire with 14% deformation has the lowest resistivity, measuring at 1.732 × 10^−8^ Ω·m, while the wire with 10% deformation has a resistivity of 1.750 × 10^−8^ Ω·m. In all three schemes, the resistivity decreases during the initial drawing phase when compared to the resistivity of the initial wire. The smaller the grain size of the metal, the more the number of internal grains and grain boundaries, and the stronger the scattering effect on electrons. Among the three different deformations, after drawing with a single-pass deformation of 14%, the internal grain size is larger, grain boundary area ratio is smaller, and electrons are less scattered when passing through the grain boundary; therefore, the resistivity is lower.

### 3.3. Influence of Different Drawing Rates on the Performance of Single-Crystal Copper Wire

The plastic deformation of the crystal is achieved by the continuous movement and proliferation of dislocations under stress. As the degree of deformation increases, the dislocation density in the crystal increases rapidly. The dislocations are entangled with each other, and the entangled dislocations form a dislocation wall; when many dislocation walls are surrounded by independent dislocation-free regions, dislocation cells are formed one by one. During the wire-drawing process, the grains are elongated along the drawing direction, and grain-structure fibrosis occurs, resulting in a larger interface area, greater resistance to dislocation movement, and improved tensile strength.

**Figure 4:**
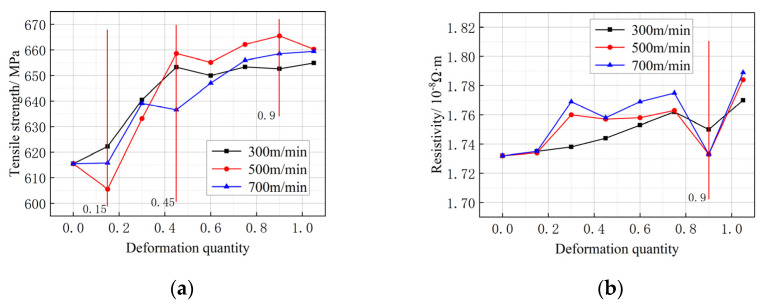
Mechanical properties and electrical conductivity of single-crystal copper wires at different drawing rates.(**a**) Tensile strength change in copper wire; (**b**) resistivity change in copper wire.

**Figure 5:**
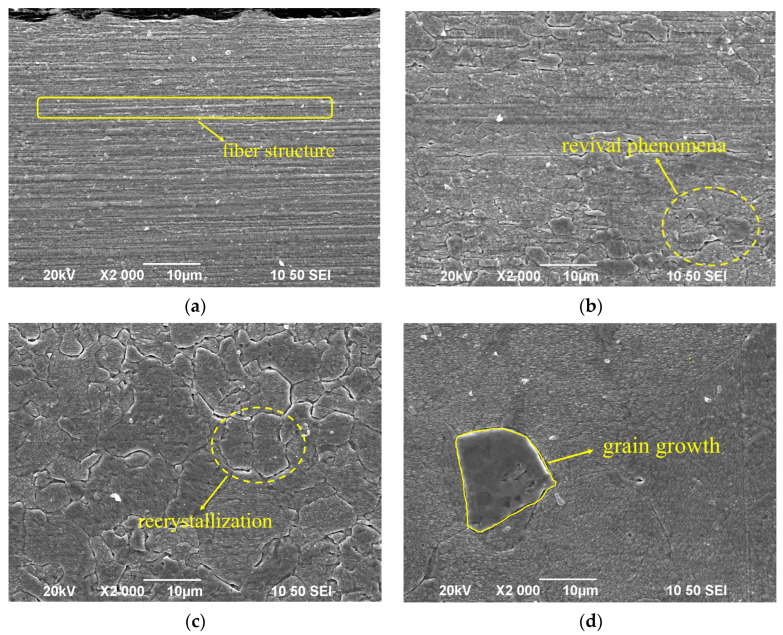
Microstructure of single-crystal copper wire under different annealing conditions. (**a**) Unannealed state; (**b**) annealing at 250 °C; (**c**) annealing at 350 °C; (**d**) annealing at 550 °C.

A Φ0.135 mm copper wire with a single-pass drawing amount of 14% was selected for the three drawing rate tests. As shown in Figure 4a, after drawing at rates of 300 m/min, 500 m/min, and 700 m/min, the tensile strength of the wire is significantly improved, compared to that of the initial wire, from 615.5 MPa to 654.9 MPa, 660.26 MPa, and 659.4 MPa, respectively. When the strain is 0.15–0.45, the tensile strength increases rapidly; when the strain is 0.45–0.9, the tensile strength tends to be stable. After drawing at 300 m/min, the elongation of the wire increases from 1.57% to 1.581%. It decreases to 1.494% and 1.39% after drawing at 500 m/min and 700 m/min, respectively. When the drawing rate is 700 m/min, it is evident that the elongation of the single-crystal copper wire decreases significantly. This decrease in elongation corresponds to a gradual increase in tensile strength and a diminishing elongation as the level of metal deformation intensifies. Figure 5b illustrates that the resistivity changes in the material under three different drawing rates are relatively minimal. Specifically, the resistivity values increase successively from 1.732 × 10^−8^ Ω·m to 1.770 × 10^−8^ Ω·m, 1.784 × 10^−8^ Ω·m, and 1.789 × 10^−8^ Ω·m, respectively. An inflection point is produced at a strain of 0.9. As the strain continues to increase, the resistivity of the material at three different drawing rates continues to increase.

### 3.4. Microstructure of Single-Crystal Copper Wires under Different Heat Treatment Temperatures

The plastic deformation of the single-crystal metal is caused by crystal slip; that is, the movement of a large number of dislocations leads to the relative movement of one part of the crystal with respect to the other part along a certain crystal plane and direction. With the continuous plastic deformation of single-crystal copper wires, the changes in the internal structure of the crystal are mainly the changes in the density, distribution, and properties of the dislocations. The shear stress required to maintain the plastic deformation will also increase with the change in the shear variables. Therefore, in order to achieve further movement of dislocations, greater stress is required; that is, work hardening occurs.

After the multipass drawing of a single-crystal copper wire, serious work hardening and residual stress is created in the copper wire. Heat treatment can partially restore the mechanical properties changed by plastic deformation, reduce or eliminate residual stress, reduce resistivity, and improve the hardness and strength of the material. Copper is an active metal that is prone to oxidation during high-temperature heat treatment. The resulting oxide layer on the surface of the bonding wire can significantly reduce the reliability of the subsequent bonding process. For copper, the oxides that react with oxygen are mainly CuO and Cu_2_O. H_2_ is a reducing gas, which can react with CuO or Cu_2_O at high temperatures, as follows:

$$CuO+H_{2}=Cu+H_{2}O$$

$$Cu_{2}O+H_{2}=2Cu+H_{2}O$$

In the heat treatment process, if H_2_ is used as the reducing gas, the products will be Cu and water vapor. Water vapor will not pollute the surface of the single-crystal copper bonding wire but will play a role in brightening heat treatment. The heat treatment temperature of the copper wire is within the range of 550 °C, and the ignition point is 572 °C, which is much lower than that of H_2_. As there is no open fire source in the heat treatment tube, the mixing of H_2_ and air in the furnace will not cause an explosion.

Figure 5a shows the microstructure of the unannealed Φ0.08 mm single-crystal copper wire; finer fibrous grains can clearly be observed. This is due to the slip phenomenon of single-crystal copper during wire drawing. Each grain is elongated along the drawing direction, following which the columnar crystal inside the wire is broken and the dislocation density increases. With the increase in strain, the broken grains gradually become orderly and elongate along the drawing direction. The degree of grain refinement continues to increase, finally forming a fibrous structure.

Figure 5b shows the microstructure of the single-crystal copper wire annealed at 250 °C. After annealing at 250 °C, the recovery phenomenon occurs in the copper wire. Owing to the movement and rearrangement of dislocations, the different dislocations on the same slip surface converge and are offset, and the fiber structure formed by grain elongation begins to gradually disseminate.

Figure 5c shows the microstructure of the single-crystal copper wire annealed at 350 °C. The deformed structure within the single-crystal copper wire due to drawing has disappeared, and the lattice defects, such as the point defects and dislocations, have undergone vigorous movement under the high temperature and have been absorbed and reduced by adjacent grains. The residual stress inside the copper wire is eliminated, the grain boundary characteristics are obvious, and the grains of the material begin to change. The new grains with low dislocation densities gradually replace the deformed grains with high dislocation density. The single-crystal copper wire now begins to recrystallize, with obvious grains appearing and gradually growing.

Figure 5d shows the microstructure of the single-crystal copper wire annealed at 550 °C. After the recrystallization of the wire, to reduce the large amount of stored energy remaining in the material, some grain sizes increase and others decrease by the movement of large-angle grain boundaries. Large grains annex small grains, resulting in grain growth. Compared to the samples annealed at 350 °C, several notable differences are observed in the samples annealed at higher temperatures, the grain size of the single-crystal copper wire annealed at 550 °C is larger, only a few larger grains can be observed in the same transverse direction, and the recrystallization process is completed.

After multipass drawing, serious work hardening and residual stress are observed in the copper wire rod. Heat treatment can partially restore the mechanical properties changed by deformation, reduce or eliminate the residual stress, reduce resistivity, and improve the hardness and strength. By eliminating the work-hardening phenomenon inside the material, the performance of the copper wire can be improved to meet the requirements of the high-performance copper wire.

### 3.5. Properties of Single-Crystal Copper Wires at Different Heat Treatment Temperatures

**Figure 6:**
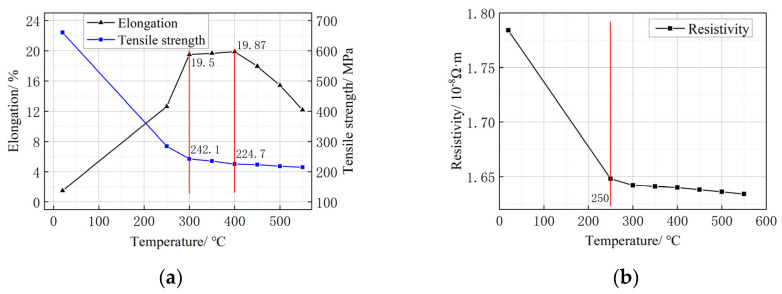
Properties of single-crystal copper wires at different annealing temperatures. (**a**) Change in mechanical properties of copper wire; (**b**) change in copper wire resistivity.

Figure 6a presents the change curve of the mechanical properties of the single-crystal copper wire at different annealing temperatures. The single-crystal copper wire without heat treatment has high tensile strength and low elongation. After heat treatment at 250 °C, the single-crystal copper bonding wire recovers, and the dislocation and residual stress inside the wire are partially eliminated and released. The tensile strength of the material decreases significantly to 284.3 MPa, which is 56% lower than that of the original sample. On the other hand, the elongation of the material increases from 1.49% to 12.6%. When the heat treatment temperature is increased to 300 °C, the tensile strength of the wire reduces to 242.1 MPa, which is 63.3% lower than that of the initial sample, and the elongation increases to 19.5%. When the annealing temperature is 400 °C, the single-crystal copper bonding wire fully recovers and recrystallizes. At this time, the tensile strength of the material reaches 224.7 MPa and the elongation becomes 19.87%. With further increase in the annealing temperature, the recrystallized grains begin to grow. In the later stages, the tensile strength of the bonding wire decreases slowly, and the elongation decreases greatly. When the heat treatment temperature is 550 °C, the recrystallized grains continue to grow, and the elongation of the wire decreases greatly. When the heat treatment temperature is 300–400 °C, the tensile strength and elongation of copper wire tend to be stable and present excellent performance.

Figure 6b presents the change in the resistivity of the single-crystal copper wire at different heat treatment temperatures. The material exhibits high resistivity before heat treatment. When the heat treatment temperature is 250 °C, the resistivity of the material reduces significantly, 1.784 × 10^-8^ Ω·m to 1.648 × 10^-8^ Ω·m. With the further increase in the heat treatment temperature, the resistivity of the material continues to decrease. When the heat treatment temperature is 550 °C, the resistivity of the single-crystal copper wire is reduced to 1.634 × 10^−8^ Ω·m. The decrease in the resistivity is mainly because of the fact that heat treatment eliminates most of the defects such as dislocations and vacancies in the material, which decreases the resistance of electron motion and the degree of scattering of free electrons, thereby increasing the conductivity.

### 3.6. Mechanical Properties and Electrical Conductivity of Single-Crystal Copper Wires at Different Heat Treatment Rates

**Figure 7:**
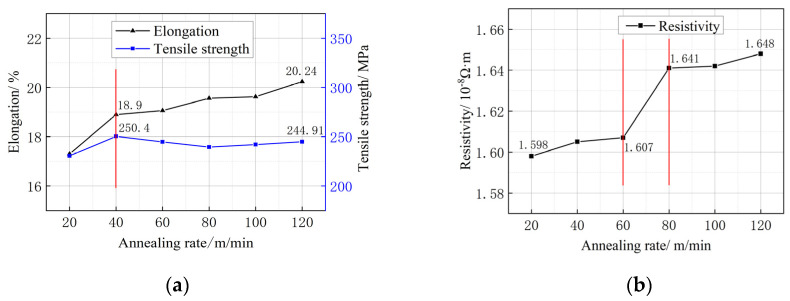
Mechanical and electrical properties of single-crystal copper wires at different annealing rates. (**a**) Change in mechanical properties of copper wire; (**b**) resistivity change in copper wire.

Figure 7a illustrates the impact of different heat treatment speeds on the mechanical properties of single-crystal copper wire at 300 °C. At an annealing rate of 20 m/min, the wire exhibits a tensile strength of 230.5 MPa and an elongation of 16.3%. As the annealing rate increases to 20–40 m/min, there is a noticeable improvement in both the tensile strength and elongation. However, further increasing the annealing rate leads to a slower increase in elongation and a slight decrease in tensile strength. At an annealing rate of 120 m/min, the wire achieves a tensile strength of 244.91 MPa and an elongation of 20.24%. Conversely, the material annealed at 20 m/min demonstrates significantly lower tensile strength and elongation compared to other annealing rates. This can be attributed to the slower annealing rate and longer annealing time, which promote a greater degree of recrystallization and grain growth within the material, resulting in a degradation of its mechanical properties.

Figure 7b depicts the resistivity change in the single-crystal copper wire at different annealing rates. Overall, the resistivity of the wire demonstrates an increasing trend as the annealing rate is increased. In the range of 20–80 m/min, the resistivity shows a slight increase from 1.598 × 10^−8^ Ω·m to 1.607 × 10^−8^ Ω·m and then from 1.607 × 10^−8^ Ω·m to 1.641 × 10^−8^ Ω·m. Finally, within the range of 80–120 m/min, the resistivity exhibits a slower increase from 1.641 × 10^−8^ Ω·m to 1.648 × 10^−8^ Ω·m. The annealing rate has a certain influence on the resistivity of the copper wire. When other conditions are fixed, a lower annealing rate is selected for annealing, and the copper wire exhibits better conductivity.

**Figure 8:**
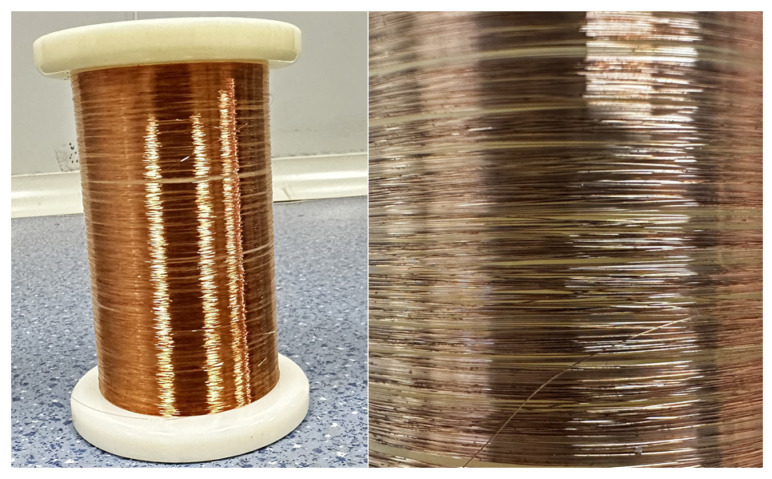
0.08 mm real wire.

Figure 8 shows a 0.08 mm wire drawn. In this paper, the properties of copper wire under different process conditions are studied. At present, the strength and elongation of the 80μ m copper wire prepared by us are high in the same diameter wire, which is conducive to the subsequent fine drawing process and provides basic guidance for the preparation of ultrafine high-performance copper wire with a diameter less than 30 μm.

## 4. Conclusions

(1) After single-pass deformations of 18, 14, and 10%, the Φ0.25 mm copper wire rod changed to a Φ0.135 mm copper wire rod, and the tensile strength increased from 500.83 to 631.3, 615.5, and 637 MPa, respectively. The single-crystal copper wire in different schemes showed a trend of rising first and then stabilizing. The elongation of the final wire was 1.47, 1.57, and 1.52%, respectively. The resistivity of the wire changed from 1.745 × 10^−8^ to 1.776 × 10^−8^, 1.732 × 10^−8^, and 1.750 × 10^−8^ Ω·m, respectively. The overall performance of the wire was the best under deformation of a single pass of 14%.
(2) The experiment involved drawing a Φ0.135 mm copper wire at rates of 300, 500, and 700 m/min, after which its tensile strength and resistivity were measured. The results indicate that both the tensile strength and resistivity increased as the wire was drawn further. Specifically, the initial tensile strength of the single-crystal copper wire was 615.5 MPa. After drawing the wire at a speed of 300 m/min, the tensile strength increased to 654.9 MPa. Subsequently, at drawing speeds of 500 and 700 m/min, the tensile strength further improved to 660.26 and 659.4 MPa, respectively. Similarly, the resistivity of the wire increased from 1.732 × 10^−8^ Ω·m to 1.770 × 10^−8^, 1.784 × 10^−8^, and 1.789 × 10^−8^ Ω·m.
(3) The Φ0.08 mm copper wire was annealed at a high temperature under an annealing rate of 100 m/min. After annealing at 300 °C, the resistivity of the wire decreased to 1.642 × 10^−8^ Ω·m. Simultaneously, the tensile strength reduced to 242.1 MPa, and the elongation increased to 19.5%. As the annealing temperature continued to increase, the tensile strength, elongation, and resistivity exhibited downward trends beyond 400 °C, and the decrease in elongation became more obvious. When the microstructure was annealed at 350 °C, the copper wire began to recrystallize. When the heat treatment temperature increased to 550 °C, the large grains swallowed up the small grains and grain growth occurred. The optimized annealing temperature of the Φ0.08 mm single-crystal copper wire was determined to be 350–400 °C.
(4) Following heat treatment of a Φ0.08 mm copper wire rod at different annealing rates, changes in tensile strength and elongation were observed. Tensile strength and elongation values were 230.5 MPa and 16.3%, respectively, at an annealing rate of 20 m/min. Meanwhile, at annealing rates ranging from 40–100 m/min, the material showed consistently high tensile strength and elongation with minor variations. At an annealing rate of 120 m/min, using single-crystal copper wire, the tensile strength was measured at 244.91 MPa, while elongation was recorded at 20.24%. However, the resistivity of this material increased from 1.598 × 10^−8^ Ω·m to 1.648 × 10^−8^ Ω·m. Overall, the single-crystal copper wire annealed within the range of 40–60 m/min exhibited excellent performance.

## Figures and Tables

**Table 1:** Drawing test schemes with different deformation.

Scheme Number	Initial Wire Diameter (mm)	Single-Pass Surface Volume Reduction (%)	Number of Molds	Drawing Speed (m/min)	Final Wire Diameter (mm)
Scheme 1	0.25	18	7	500	0.135
Scheme 2		14	9		
Scheme 3		10	12		

**Table 2:** Drawing test schemes with different rates.

Scheme Number	Initial Wire Diameter (mm)	Single-Pass Surface Volume Reduction (%)	Drawing Speed (m/min)	Final Wire Diameter (mm)
Scheme 1	0.135		300	0.08
Scheme 2		15	500	
Scheme 3			700	

**Table 3:** Annealing test scheme.

Equipment Type	Initial Wire Diameter (mm)	Annealing Temperature (°C)	Annealing Rate (m/min)
SD-40	0.08	250–550	500
SD-40	0.08	300	20–120

## Data Availability

The data presented in this study are available on request from the corresponding author. The data are not publicly available due to the partner needs to keep the original data confidential, it cannot be shared.
